# Harnessing the Power of *Alchemilla*: A Natural Solution for Skin Health and Dermatological Disorders

**DOI:** 10.3390/molecules30081861

**Published:** 2025-04-21

**Authors:** Sebastian Kanak, Barbara Krzemińska, Anna Berecka-Rycerz, Monika Kopeć, Katarzyna Dos Santos Szewczyk

**Affiliations:** 1 Department of Pharmaceutical Botany, Medical University of Lublin, Chodźki 1, 20-093 Lublin, Poland; sebastian.kanak@umlub.pl (S.K.); barbara.krzem@gmail.com (B.K.); 2 Department of Medicinal Chemistry, Medical University of Lublin, Jaczewskiego 4, 20-090 Lublin, Poland; anna.berecka-rycerz@umlub.pl; 3 Chair and Department of Obstetrics and Perinatology, Medical University of Lublin, Jaczewskiego 8, 20-090 Lublin, Poland; monika.ruszala@wp.pl

**Keywords:** *Alchemilla*, Rosaceae, skin disorders, plant extracts, natural agents, anti-aging

## Abstract

Skin diseases are prevalent and encompass a wide range of disorders with varying clinical manifestations and diverse etiopathogenesis. The response to the necessity of multidirectional treatment is provided by species belonging to the genus *Alchemilla*, which is used in traditional medicine as well as in cosmetic formulations. Responsible for the healing properties of these plants for skin diseases are mainly compounds such as tannins, phenolic acids, flavonoids, and anthocyanins. The aim of the study was to analyze and synthesize the published literature on the *Alchemilla* species in skincare with a timeframe of December 2024. The literature indicates that due to antioxidant, anti-inflammatory, astringent, antimicrobial, elastase activity and tyrosinase inhibitory properties of various *Alchemilla* species, extracts obtained from these plants can be successfully applied in skin rashes, acne, stretch marks, eczema, psoriasis, wrinkles and other dermatological issues. To the best of our knowledge, this is the first review of the cosmetic activities of *Alchemilla* species.

## 1. Introduction

For millennia, medicinal plants and their phytochemicals have been integral to traditional medicine across cultures, including the treatment of skin ailments [1]. Ancient healers in systems from Ayurveda to European folk medicine used herbal preparations for wound care, inflammation, and skin rejuvenation, laying the groundwork for modern phytotherapy [1]. This historical reliance on botanicals is attributed to the presence of bioactive compounds (e.g., polyphenols, tannins, essential oils) that provide therapeutic effects such as antiseptic, anti-inflammatory, and wound-healing actions [1]. These early observations have inspired contemporary research into plant-derived compounds for dermatological uses, with a renewed interest in natural, safer alternatives to synthetic drugs.

In recent years, factors like increasing pollution and consumer demand for “green” skincare have caused a revival in plant-based dermatological products. Botanical extracts offer multidirectional benefits due to the synergism of their constituents, an advantage in managing complex skin disorders that often involve oxidative stress, microbial infection, and inflammation [2]. The genus *Alchemilla* (Rosaceae), commonly known as lady’s mantle, is an example of such a plant resource. *Alchemilla* spp. are perennial herbs (about 795 accepted species worldwide) traditionally used as healing agents for numerous conditions, including dermatological issues [3]. Ethnobotanical records confirm their use for treating wounds and abrasions—for instance, *A. hessii* in Turkey is applied to cuts [4], and *A. monticola* has been used for wounds and burns in Europe [5]. These folkloric uses suggest *Alchemilla* plants possess wound-healing and anti-infective properties.

**Figure 1:**
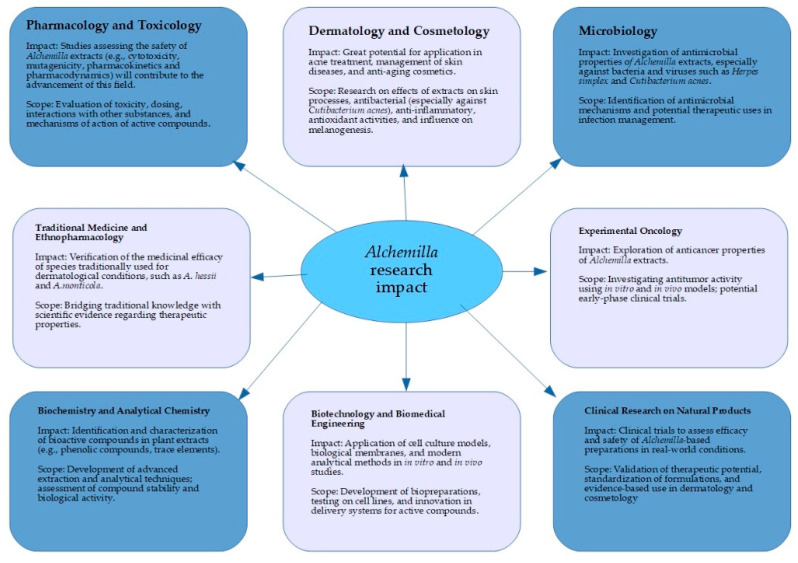
*Alchemilla* research impact.

*Alchemilla* species are rich in polyphenolic compounds (tannins, flavonoids, phenolic acids, anthocyanins) believed to be responsible for their skin-healing effects [6]. However, a comprehensive evaluation of their dermatological benefits has been lacking. Therefore, the aim of this review is to synthesize current knowledge on the cosmetic and dermatological activities of *Alchemilla* species, including their antioxidant, anti-inflammatory, antimicrobial, anti-aging, and wound-healing properties, and to discuss practical applications and future research directions (Figure 1). To our knowledge, this is the first focused review of *Alchemilla* in skin care and dermatological therapy, highlighting its potential as a multi-functional natural agent.

## 2. Methodology of Evidence Acquisition

**Figure 2:**
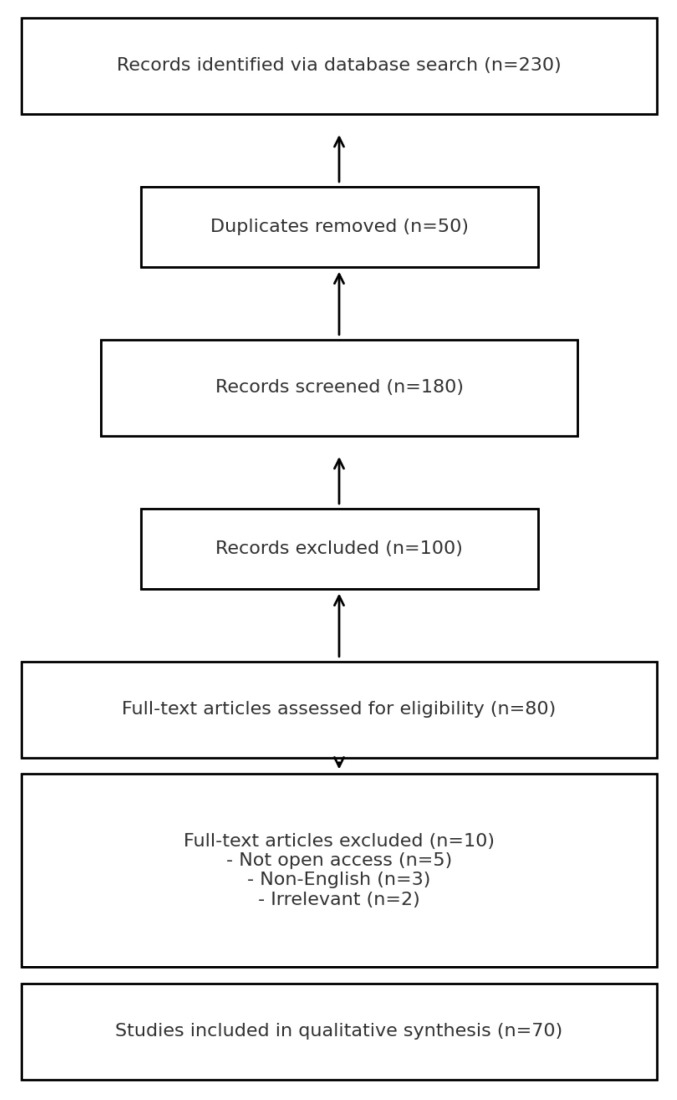
PRISMA flow diagram of the literature search and selection (adapted from [7]). Out of 230 records identified, 70 studies were included in this review after screening and eligibility assessment.

This review analyzed the literature on *Alchemilla* and skin-related applications up to December 2024. Multiple databases were searched (Google Scholar, Scopus, Web of Science, ResearchGate, Medline, SciFinder, ScienceDirect, PubMed) using keywords such as “*Alchemilla* skin”, “*Alchemilla* antioxidant”, “*Alchemilla* anti-inflammatory”, “lady’s mantle cosmetic”, and related MeSH terms. Inclusion criteria were English-language publications of original studies directly relevant to *Alchemilla* and dermatology. Priority was given to peer-reviewed articles and highly cited papers; however, due to the novelty of this topic, a few conference abstracts (with DOIs) and a patent were included for completeness. Studies where *Alchemilla* was a component of a multi-herb mixture were included if results for *Alchemilla* could be discerned. Exclusion criteria included non-English articles, clearly irrelevant works, duplicate publications, and sources inaccessible online. Using this strategy, we initially identified 230 records. After removing duplicates, 180 unique records remained for screening. Title and abstract screening excluded 100 records that did not meet the inclusion criteria (e.g., unrelated to skin or lacking experimental data). We retrieved 80 full-text articles for detailed assessment, of which 10 were excluded (5 not accessible in full text, 3 non-English, 2 lacking dermatology-related outcomes). In total, 70 publications were included in this review for qualitative synthesis. Figure 2 presents the study selection process in a PRISMA 2020 flowchart, following Page et al. [7] guidelines. The included studies encompass in vitro assays, in vivo animal experiments, and a few clinical or ex vivo investigations, providing a broad evidence base for *Alchemilla*’s dermatological effects. Out of 230 records identified, 70 studies were included in this review after screening and eligibility assessment.

## 3. Biological Activities of Alchemilla Species Towards Skin

**Figure 3:**
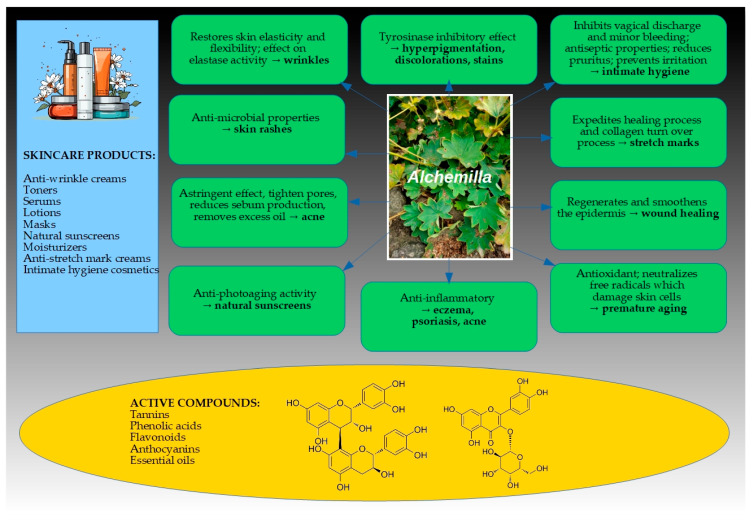
Biological activities towards skin disorders and the applicability of extracts obtained from different *Alchemilla* species in the cosmetic industry.

As shown by the gathered evidence, various *Alchemilla* extracts demonstrate a range of beneficial biological activities relevant to skin health (summarized in Table 1 and Figure 3). Key activities include antioxidant effects, anti-inflammatory activity, antimicrobial (antibacterial and antifungal) properties, anti-aging effects (e.g., inhibition of collagenase, elastase, and tyrosinase enzymes), wound-healing promotion, and even anti-tumor effects on skin cancer cells. These activities, discussed below, underlie the traditional uses of *Alchemilla* in treating rashes, acne, eczema, wounds, and other skin ailments. Table 1 organizes the findings by biological activity, summarizing the *Alchemilla* species studied, extract types, experimental models, and main results for each category.

### 3.1. Antioxidant Activity

Secondary metabolites such as phenolic compounds of plant origin are a promising tool for preventing skin damage. Increased levels of ROS and oxidative stress lead to the loss of cellular redox homeostasis and, consequently, to damage to proteins, DNA and lipids through various signaling pathways. Reduction of fibroblast functionality contributes to the appearance of clinical features of aging. Normal fibroblast function and increased collagen expression can be restored by treatment with antioxidants such as polyphenols [42]. Oxidative stress contributes to the modification of several biomolecular pathways in cells. Photoaging is connected with MAPKs/AP-1, MMP-1/3/9, p-c-Fos, p-c-Jun pathways, abnormal pigmentation is associated with melanogenic paracrine mediators, tyrosinase and tyrosinase-related protein-1 (TRP-1), while inflammation is generated via p-ERK ½ MAPK, NF-kB, inflammatory cytokines (IL-6, TNFα, IFN-γ) [43]. A study conducted by Garcia-Villegas et al. [44] showed that sometimes, even inconspicuous plant parts, such as cherry stems, may turn out to be a valuable therapeutic raw material for antioxidant activity through cosmetic applications. A comprehensive analysis of the chemical composition using HPLC-ESI-qTOF-MS showed the presence of 146 chemical compounds, including these valuable in the treatment of dermatological disorders, and high values of antioxidant properties in FRAP, TEAC, and ORAC assays. In addition, seemingly well-researched and widely used black tea (the extract obtained through plant small RNA PSRTM technology) has shown excellent anti-aging properties due to its ability to affect the mitochondria, nuclear DNA integrity as well as the extracellular matrix [45]. The above-mentioned examples confirm the necessity to study previously untested species, including so far unexplored *Alchemilla* species.

Among all biological activities, antioxidant properties are most extensively researched in the entire *Alchemilla* genus, and at the same time, this activity is valuable from a cosmetic point of view, which makes this plant material worthy of attention. Interestingly, although antioxidant activity is the most extensively studied property among the genus *Alchemilla*, it turns out that among 795 accepted species, only 12 species have been studied towards this capacity. Methanolic extracts of *A. acutiloba* (and their ethyl acetate and butanol fractions) showed potent free-radical scavenging in DPPH and ABTS assays, quenching ~95% of radicals at 50 µg/mL [8]. The ethyl acetate fraction of aerial parts was especially active (94.8% DPPH scavenging) [8]. Methanol extract of aerial parts of *A. alpina* displayed high DPPH scavenging (45.4% to 94.4% inhibition, depending on concentration) [9], indicating significant antioxidant capacity. Among methanol, hexane, acetone, and water extracts of *A. arvensis* tested, the acetone extract had the strongest DPPH radical scavenging (IC_50_ = 4.9 µg/mL) [10], outperforming other solvents. This suggests moderately polar solvents extract the most active antioxidant compounds. Crude methanolic extract and its fractions (hexane, chloroform, water) from *A. barbatiflora* were evaluated by DPPH, superoxide radical (SOD) scavenging, phosphomolybdenum, and FRAP assays. The methanol extract, and especially its aqueous fraction, showed the highest antioxidant activities [11], highlighting that polyphenols concentrated in polar fractions confer strong antioxidative effects. A leaf hexane extract of *A. ellenbergiana* exhibited very potent DPPH scavenging (IC_50_ = 7.1 µg/mL) [12]. However, ethanol and methanol extracts of this species were less active (IC_50_ = 243 µg/mL) [13], suggesting that specific non-polar constituents (possibly certain flavonoids or other phenolics) in *A. ellenbergiana* contribute to its antioxidant effect. An 80% acetone (in 0.2% formic acid) extract of *A. glabra* demonstrated significant antioxidant capacity in ORAC, TRAP, and HORAC assays [14]. An ethanolic extract of *A. hybrida* also showed strong antioxidant and radical-scavenging effects (DPPH IC_50_ ~ 0.082 mg/mL) [15]. This extract also contained high total phenolics, correlating with its potent activity.

### 3.2. Anti-Inflammatory Activity

The anti-inflammatory effect of plant extracts is particularly important in the case of chronic inflammatory skin diseases such as psoriasis and atopic dermatitis, in which increased expansion of inflammatory cells and hyperproliferation of keratinocytes are observed. The main focus of active plant extracts is the mTOR (mechanistic target of rapamycin) pathway, which can control the activation and differentiation of keratinocytes. Interestingly, keratinocytes express high levels of glucose transporter (GLUT)1, L-type amino acid transporter (LAT)1, and cationic amino acid transporters (CATs) [46].

Several *Alchemilla* extracts can mitigate inflammation, often assessed via enzyme inhibition or cell-based models.

Butanol and ethyl acetate fractions (50 µg/mL) from *A. acutiloba* showed notable inhibition of pro-inflammatory enzymes cyclooxygenase-1 and -2 (COX-1, COX-2) [8]. In vitro assays indicated COX-2 inhibition up to ~80% by the butanol fraction of aerial parts, comparable to standard anti-inflammatory agents. This suggests *A. acutiloba* contains constituents (likely polyphenols or tannins) that interfere with inflammatory eicosanoid synthesis.

Oral supplementation with *A. mollis* extract in mice has demonstrated anti-inflammatory and anti-photoaging effects. In a UVB-induced skin damage model, dietary *A. mollis* protected against inflammation by downregulating NFATc1 and upregulating Nrf2/ARE pathways, thereby reducing cytokine release and oxidative stress in the skin [18]. This in vivo result aligns with the traditional use of *A. mollis* for soothing skin irritations.

As reported by Oz et al. [20], methanolic extracts of *A. persica* and *A. mollis* in the Whittle method (acetic acid-induced increase in capillary permeability on a group of 10 mice in 0.2 mL/20 g body weight) revealed anti-inflammatory effect at concentration 26.6% at 200 mg/kg dose and 30.6% at 200 mg/kg dose, respectively. Kurtul et al. [32] performed a study using the human red blood cell (HRBC) membrane stabilization method (measuring stabilization capacity against heat-induced hemolysis of HRBC membrane). In this trial, the methanolic extract of *A. mollis* aerial parts revealed the highest anti-inflammatory activity, with IC_50_ = 1.22 mg/mL. For a methanolic extract of *A. persica*, the IC_50_ value for aerial parts extract was 1.52 and in the case of roots, 1.82 mg/mL.

It is worth noting that agrimoniin isolated from *Alchemilla* (e.g., in *A. mollis* extract) has shown anti-inflammatory activity in some studies [47]. Hoffmann et al. [48] demonstrated that agrimoniin possesses significant anti-inflammatory effects, which likely contribute to Alchemilla’s activity when present.

### 3.3. Antibacterial and Antifungal Activity

The rising phenomenon of resistance, as well as unexpected side effects of conventional pharmaceutical drugs, have caused scientists to turn to the plant kingdom as a potential source of new antimicrobial agents. The antibacterial effect of extracts applied to the skin is important because various diseases (such as acne eruptions) result in wounds and associated skin superinfections. According to the latest knowledge, not only the antimicrobial effect but also the interactions of various extracts and other cosmetic ingredients with the network of the skin microbial ecosystem are of significant importance [49].

*Alchemilla* extracts exhibit broad antimicrobial effects, particularly against Gram-positive skin pathogens, which supports their use in treating infected wounds and acne.

Methanolic, hexane, acetone, and water extracts from *A. arvensis* were tested against bacteria, including *Staphylococcus aureus*, *Escherichia coli*, *Pseudomonas aeruginosa*, and *Shigella sonnei*. All extracts showed antibacterial activity to varying degrees. Notably, S. aureus (a common cause of skin infections) was inhibited by all extracts (inhibition zones 10–20 mm) except a lack of activity against *P. aeruginosa* [10]. MIC values ranged from 6.3 to 25 mg/mL for most bacteria, with *P. aeruginosa* being the least susceptible (no inhibition by some extracts). These results highlight *A. arvensis* potential as a source of topical antibacterial agents, especially for Gram-positive organisms.

Various extracts of *A. mollis* demonstrated significant antibacterial potency. A 50% methanolic extract had MICs of 0.5 mg/mL against *S. aureus*, 2.0 mg/mL against *P. aeruginosa*, and 5.0 mg/mL against *Enterococcus faecalis* [19]. The aqueous and deodorized water extracts showed similar MIC profiles (e.g., 0.5 mg/mL for *S. aureus*). The consistently low MIC for *S. aureus* across extract types indicates *A. mollis* is particularly effective against this bacterium. These findings are promising for developing *A. mollis*-based treatments for staphylococcal skin infections.

Interestingly, although the *Cutibacterium acnes* bacterial strain (formerly called *Propionibacterium acnes*) plays a pivotal role in the etiopathogenesis of *acne vulgaris*, and what is more, the prevalence of acne is very common [50], in the scientific literature, there is only one publication regarding the activity of *Alchemilla* against this strain, i.e., Dzabijeva et al. [34] tested ethanolic extract from *A. vulgaris* against *P. acnes* subsp. acnes MSCL 1521 (ATCC 6919) and *Propionibacterium granulosum* MSCL 1522 (ATCC 25564) using the agar well diffusion method. The obtained diameter of inhibitory zones was 10 mm for *Propionibacterium acnes* and 13 mm for *P. granulosum*. At the same time, in this study, the ethanol extract of *A. vulgaris* turned out to be inactive against *S. epidermidis* [34]. This suggests some efficacy in targeting the anaerobic bacteria involved in acne lesions, which, combined with *Alchemilla*’s anti-inflammatory tannins, could benefit acne treatment. Given the prevalence of acne, further testing of other active species (e.g., *A. mollis*, *A. arvensis*, *A. pedata*) against *C. acnes* is warranted, as they have shown substantial antibacterial effects in general.

As reported by Taddese et al. [33], formulations with 10% of *A. pedata* extract have been assessed for their ability to inhibit *E. coli*, *P. aeruginosa* and *S. aureus* growth. In the case of formulation 1 (extract in Sodium laurylmonosterin cream base), form 2 (in Macrogol cream base), form 3 (in Macrogol blend Ointment base), form 6 (in Polyethylene glycol bases PEG 400:PEG 2000, 1:1) and form 7 (in Polyethylene glycol bases PEG 400:PEG 2000, 1:3) the following MIC values were obtained: *E. coli*: 14.3–16.2 mg/mL, *P. aeruginosa* 14.0–23.5 mg/mL and *S. aureus* 17.5–22.5 mg/mL. At the same time, formulations 4 (extract in simple ointment base) and 5 (white petrolatum) turned out to be inactive. In the study conducted by Boroja et al. [26], *A. vulgaris* extracts showed significant activity against *Enterococcus faecalis*, *Salmonella typhimurium*, *Micrococcus lysodeikticus* and *Bacilus mycoides* with MIC values 0.156–0.625 mg/mL. In summary, only the following *Alchemilla* species have been tested for antibacterial activity: *Alchemilla arvensis*, *A. mollis*, *A. pedata*, *A. persica* and *A. vulgaris*.

Taking into consideration that fungal infections of the skin (including dandruff, oral candidiasis, onychomycosis, and mycosis fungoides of the feet) are very common, the exploration of new substances derived from plants that could support their treatment is an extremely relevant research direction. Moreover, the search for active antifungal substances with a high safety profile has become an important goal of the cosmetics industry. Nevertheless, studies performed so far prove the negligible activity of *Alchemilla* species against *Candida albicans*.

The same *A. arvensis* extracts tested for bacteria were evaluated against fungi such as *C. albicans* and *Epidermophyton floccosum*. The results showed antifungal activity with MICs of 3.7–12.5 mg/mL for *C. albicans* and 0.78–6.3 mg/mL for *E. floccosum* [10]. These ranges suggest that E. floccosum (a dermatophyte causing skin infections) is more susceptible to *A. arvensis* extracts than *Candida*. This antifungal efficacy supports the traditional use of *Alchemilla* for treating rash and eczema, conditions where secondary fungal infections can occur.

An ethanolic extract of *A. hybrida* not only was a strong antioxidant but also exhibited antifungal effects. It inhibited the growth of various fungi with minimum inhibitory concentrations ranging from 0.104 to 1.667 mg/mL and minimum fungicidal concentrations of 0.208 to 2.5 mg/mL [15]. These values (from microdilution assays) indicate a broad-spectrum fungistatic effect, with a complete fungal kill at slightly higher concentrations. The combined antioxidant and antifungal *A. hybrida* profile suggests it could protect the skin by both reducing oxidative stress and directly suppressing fungal pathogens.

*A. pedata* showed no activity against *C. albicans* in the study conducted by Taddese et al. [33]. Furthermore, *A. mollis* was not active towards this strain in the analysis carried out by Karatoprak et al. [19]. The methanol extract of aerial parts and roots of *A. vulgaris* had no inhibition effect on the growth of *C. albicans* in the survey performed by Boroja et al. [26]. Nonetheless, the same analysis showed the activity of *A. vulgaris* extracts against other fungal strains, namely *Phialophora fastigiata*, *Penicillium canescens*, *Trichoderma viride*, *Trichoderma longibrachiatum*, *Aspergillus brasiliensis*, *Aspergillus glaucus*, *Fusarium oxysporum*, *Alternaria alternata* and *Doratomyces stemonitis* (MIC values ranged from 2.5 to 20 mg/mL). All tested extracts showed weaker antifungal activity than nystatin, which showed activity at concentrations from 0.078 to 5 µg/mL [26].

### 3.4. Wound Healing Activity

The issue of searching for therapeutic agents with the potential to treat wounds is important, among others, because wounds occur in the course of dermatoses such as acne, eczema or psoriasis. Fibroblasts are relevant in the wound healing process because they proliferate and migrate to the injured area, synthesizing new extracellular matrix. Moreover, keratinocytes re-differentiate and restore the normal epidermal barrier. In the process of wound healing, plant extracts contribute to the suppression of protease activity, which creates a normal environment and restores normal skin functions. Moreover, they enhance angiogenesis quickly by responding to increased macrophage migration and metabolic demand for new tissue [37]. Significant progress in knowledge over the last few years regarding the healing mechanism at the molecular level has allowed scientists to replace experimental animal models with alternative in vitro models. Among such models is an in vitro test based on a monolayer of fibroblasts with artificially formed wounds, which is of great importance. A multidirectional and innovative approach to wound treatment in designing medicinal formulations, taking into account the current understanding of the molecular and cellular basis of tissue healing processes, is of great importance. Different phases of wound healing can be distinguished, namely clot production, re-epithelialization, angiogenesis, and scar deposition. It is well established that keratinocytes, fibroblasts, melanocytes, adipocytes and cutaneous nerves are relevant in this process [51].

A few *Alchemilla* species have been investigated for their ability to accelerate wound closure and skin regeneration, validating their use in folk medicine for wounds.

Tasić-Kostov et al. [35] conducted an extensive study on the effects of *Alchemilla vulgaris* on wound healing processes using in vitro wound healing assay with L929 fibroblasts and in vivo assessment of skin barrier repair potential. The most beneficial effect on fibroblast migration as well as wound healing in vitro was observed for ethanolic extract, which is most likely related to the rich chemical composition of this extract determined by RP-HPLC, i.e., ellagic acid 3.4 mg/g, isoquercetin 4.0 mg/g. What is more, gel with propyleneglycolic extract revealed the most favorable effect on in vitro wound healing and showed the highest value of adhesiveness in TPA—Texture profile analysis (Texture Analyser). In vivo, tests were carried out on 18 volunteers using Corneometer^®^ CM 820 (measurements of stratum corneum hydration on SLS-irritated human skin) and Tewameter^®^ TM 210 (measurements of transepidermal water loss). Gels containing *A. vulgaris* extract revealed significant improvement in the skin hydration level after 3 days of application. Interestingly, after 7 days, stratum corneum hydration was even higher than baseline. These results from Tasić-Kostov et al. demonstrate that *A. vulgaris* facilitates the healing process, likely due to its tannins creating a protective layer and its phytochemicals stimulating tissue repair.

Another study that examined the wound-healing activity of extracts obtained from *A. vulgaris* was conducted by Shrivastava et al. [36]. The effect of *A. vulgaris* on the growth of epithelial cells and myofibroblasts was tested in Chang liver and Madin Darby Bovine Kidney (MDBK) epithelial cell lines and rat aortic myofibroblast cultures. *A. vulgaris* extract at concentration 0.1–1.0% caused a significant increase in cell numbers, namely 21.3% in MDBK, 15.5% in myofibroblast and 10.6% in Chang liver cells. Treatment of rats with the 3% *A. vulgaris* extract resulted in a reduction in the diameter of lesions by 10.0 7% after 2 days and 23.2% after 3 days.

A herbal mixture containing *A. vulgaris* extract (20%), *Mimosa tenuiflora* leaf extract (20%), Glycerol (42%), Honey (10%), and Xanthan Gum (8%) was tested in vivo using a wound model on seven-week-old male BALB/c mice (macroscopic analysis, immunostaining and confocal microscopy) as well as in vitro (scratch assay). The tested plant mixture showed a significant ability to support wound healing [37].

In an in vivo excision and incision wound model on male Swiss albino mice and Sprague-Dawley rats, *A. mollis* methanolic extract showed a remarkable wound healing effect. Treated wounds had ~39% greater tensile strength than controls and significantly faster contraction (51% wound closure) after a set period [20]. Histological analyses confirmed enhanced granulation tissue formation. These results, reported by Oz et al., highlight *A. mollis* as a potent wound healer, validating its traditional use on cuts. The same study found *A. persica* extract improved wound healing, though slightly less than *A. mollis*, with a 33% increase in tensile strength and 43.5% wound contraction [20]. Both *A. mollis* and *A. persica* accelerated healing compared to untreated wounds, suggesting that multiple *Alchemilla* species contain bioactives (likely tannins and flavonoids) that promote collagen deposition and wound closure.

### 3.5. Anti-Aging and Skin-Lightening (Enzyme Inhibitory) Activity

The skin is the largest organ of the human body (the total surface area is 1.5–2 m^2,^ and the thickness is 1.5–5 mm) and has a complex structure since it consists of three layers: epidermis, dermis (mainly composed of fibroblasts producing collagen) and subcutaneous tissue [42,52]. The Extracellular Matrix (ECM) of the skin is an organized network of insoluble macromolecules that constitute a physical scaffold for cells. It provides unique skin properties, such as elasticity, compressibility and high tensile strength. It consists of polysaccharides, collagen proteins (collagen, elastin—they are responsible for the ECM elasticity), proteoglycans (dermatan sulfate and hyaluronan—they cush-ion cells in the extracellular matrix), hyaluronic acid and water. ECM molecules secreted by fibroblasts and epidermal cells are of great importance [38]. Numerous factors, both external and internal, contribute to the acceleration of the skin aging process. Thus, the following actions should be taken: inhibiting the elastase and collagenase enzymes, decelerating negative changes in fibroblasts, and reducing the amount of free radicals using free radical scavengers.

Skin aging is associated with loss of elasticity, caused by changes in elastin—a protein that forms connective tissue together with collagen and glycosaminoglycans. Miscellaneous factors such as, among others, pregnancy, cellulite, rapid changes in body weight, solar radiation and air pollution affect the connective tissue and contribute to the loss of skin elasticity. Moreover, skin disorders such as psoriasis, contact dermatitis, and atopic dermatitis cause increased elastase activity and degradation of elastin fibers. Enzymes such as elastases (belonging to the chymotrypsin family of proteases) and collagenases are responsible for the breakdown of elastin and collagen. Scientists are still looking for active ingredients that effectively inhibit the elastase and collagenase enzymes with a significant safety profile [2].

*Alchemilla* extracts have shown the ability to inhibit enzymes that contribute to skin aging and hyperpigmentation, aligning with their use in anti-aging cosmetics. However, only *A. vulgaris* has been tested for its ability to inhibit these enzymes. Some studies indicate that *A. vulgaris* contains compounds that inhibit collagenase, elastase, and tyrosinase—enzymes responsible for collagen breakdown, loss of skin elasticity, and melanin overproduction. As reported by Mandrone et al. [38,39], methanolic extract of *A. vulgaris* at a concentration of 50 µg/mL exhibited collagenase inhibition due to the presence of such compounds as tannins or quercetin-3-*O*-*β*-glucuronide. The plant complex containing *A. vulgaris*, *Silybum marianum*, *Equisetum arvense*, *Glycine soja*, *Triticum vulgare*, *Medicago sativa* and *Raphanus sativus* in the test of enzymatic inhibition using enzymes porcine pancreatic elastase (PPE) and human leukocyte elastase (HLE), revealed significant inhibition in the order of 41.0% against PPE and 50.0% against HLE [2]. In the same study, in vivo and in vitro tests using the SEM 474 Cutometer (Courage & Khazaka) revealed that applying a cosmetic formulation containing 5% of the plant complex caused a significant increase in skin elasticity [2]. According to Chiocchio et al. [40], methanolic extract of *A. vulgaris* at 50 μg/mL showed 12% elastase inhibitory activity, correlated to the total phenolic and flavonoid content.

Another enzyme involved in the aging process is tyrosinase. Tyrosinase (polyphenol oxidase—PPO) is an enzyme including copper, playing a vital role in two reactions: the conversion of an o-diphenol to the o-quinone as well as the hydroxylation of a monophenol. Furthermore, tyrosinase participates in the biosynthetic pathway of melanin. Thus, it is associated with age-related skin hyperpigmentation. Tyrosinase inhibitors reveal skin-whitening properties [40].

Vlaisavljevic et al. [27] conducted a study assessing the ability of *Alchemilla vulgaris* methanolic, ethanolic, ethylacetate and water extracts to inhibit tyrosinase. The best activity was observed for methanolic, followed by ethanolic, ethylacetate and water extracts (79.84 mg KAE per g for methanolic extract; the enzyme inhibitor effect was evaluated as equivalents of kojic acid—KAE). The compounds that are largely responsible for this effect are apigenin-7-*O-β*-glucoside, luteolin-7-*O-β*-glucoside, quercetin and rutin. Moreover, ethanolic and water extracts of *A. vulgaris* were evaluated spectrophotometrically using l-DOPA as a substrate for their tyrosinase inhibition [25]. Ethanolic extract (3 mg/mL) revealed significant activity (71.55), mainly due to the presence of polyphenols (112.33 GAE μg/mL), proanthocyanidins (130.00 catechine μg/mL) and flavones (497.00 rutin μg/mL). In the discussed publication [25], HPLC analysis showed that the 70% ethanol extract of *A. vulgaris* contains large amounts of ellagic acid (996.6 μg/mL), which, according to Ortiz-Ruiz et al. [53], is a tyrosinase inhibitor and is used in the cosmetic industry as a whitening agent. The complex of Swiss Alpine plant extracts containing *A. vulgaris* significantly reduced the color intensity of dark spots among human volunteers (measured by solar simulator equipment and light meter reading) [54]. On the other hand, *A. vulgaris* methanolic extract showed no tyrosinase inhibitory activity tested at 50 μg/mL in *an* in vitro tyrosinase inhibitory assay [40]. It is worth noting that the only species of the *Alchemilla* genus that has been tested for tyrosinase inhibition is *A. vulgaris*, which provides a large space for further research.

Natural skin aging is associated with changes in peripheral microfibrils. Namely, their number decreases, they become thicker, the surface of these microfibers becomes irregular and granular, and the number of glycosaminoglycans and fibroblasts reduces. External aging is associated with the presence of amorphous elastin fibers and an increase in cross-links between the fibers. The normal functionality of elastin is prevented due to the increase in the number and changes in glycosaminoglycans, which reduces the functionality of fibroblasts. Fibroblasts create a structural scaffold for tissues, maintain the integrity of connective tissue, release elastic fibers, and, therefore, prevent signs of aging [42].

A study conducted by Borodušķis et al. [55] showed the effect of a complex of seven plant extracts (brand name Cell Repair Bio-Complex CRB), including *A. vulgaris*, on the proliferation of dermal fibroblasts and keratinocytes (using the IncuCyte ZOOM system). Another species of *Alchemilla* showing a beneficial effect on normal human dermal fibroblasts (NHDF) due to AP-1 and Nrf2/ARE signaling pathways is *A. mollis* (ethanolic extract, abundant in gallic acid) [18].

### 3.6. Protective Effect Against UVB Radiation

UV rays, in addition to their beneficial properties, such as the production of vitamin D in the skin, have many negative consequences related to the condition of the skin. In particular, they significantly accelerate the skin aging process and lead to the development of carcinogenesis, which may result in melanoma. Phytochemicals due to their multidirectional activity reveal a wide range of photoprotective effects, including anti-photoaging, anti-inflammation, and anti-melanogenesis and via activation of nuclear factor erythroid 2-related factor 2 related to the antioxidant response element (Nrf2-ARE) signaling pathway, regulating antioxidant genes (including glutamate cysteine ligase (GCL), glutathione S-transferase (GST), NAD(P)H quinone oxidoreductase-1 (NQO-1), heme oxygenase-1 (HO-1)) [43].

Plants of the genus *Alchemilla* are used in traditional medicine for skin dysfunctions related to excessive exposure to sunlight. Ethnobotanical surveys conducted by Mrabti et al. [56] showed that the powder of leaves obtained from *Alchemilla vulgaris* (local name Gdam sbaa) is used in traditional medicine in the Taza region in Morocco for burns and, therefore, has significant anti-burn activity. Protective effects of ethanolic extract obtained from *Alchemilla mollis* against extrinsic aging caused by UVB radiation were conducted in vitro on UVB-irradiated normal human dermal fibroblasts (NHDF) and hairless mice skin in vivo. The study revealed a significant anti-photoaging effect connected with AP-1 and Nrf2/ARE signaling pathways. Most likely, this effect is related to the presence of significant amounts of gallic acid in the extract [18].

A major direction of research is the search for substances that eliminate melanoma, the main cause of which is overexposure of the skin to the sun’s rays. Of course, treatment with plant agents will not replace treatment with traditional chemical drugs, but it can significantly support them due to the multidirectional action of plants. Jelaca et al. [41] carried out a study on the effect of ethanolic extract of *A. vulgaris* against melanoma cells in vitro and in vivo, as well as its effect on tumor microenvironment ex vivo. For this purpose, the following mouse melanoma cell lines were used: B16 and B16F10. Moreover, an in vivo syngeneic mouse melanoma model was employed. The results revealed a dose-dependent decrease of cell viability after 72-h treatment with *A. vulgaris* extract and significantly reduced tumor growth in the B16 melanoma model in vivo [41].

### 3.7. Moisturizing Properties

Water is a substrate as well as a product of various reactions and a relevant ingredient that plays a significant role in the proper functioning of the skin, especially in the stratum corneum, where it combines with lipids and proteins. In corneocytes, i.e., dead cells of the stratum corneum, proteins adhere tightly to each other. Moreover, they are surrounded by a lipid matrix, which constitutes a specific skin barrier that prevents excessive transepidermal water loss (TEWL). Moreover, The Natural Moisturizing Factor (NMF), which contains free amino acids, pyrrolidone carboxylic acid (PCA), trans urocanic acid (UCA), sugars and lactic acid, is a natural skin protector against dehydration [57].

The study conducted by Załęcki et al. [57] showed that both water consumption as well as physical activity are positively correlated with skin hydration. Topical cosmetic preparations should also be used to maintain skin hydration at a satisfactory level in addition to these types of health-promoting activities. Extracts obtained from *A. vulgaris*, whose moisturizing properties have been scientifically proven, maybe the answer to the need for topical cosmetic application of cosmetic formulations. Other *Alchemilla* species have not yet been tested for their ability to moisturize the epidermis.

Analysis related to the effect of gels with ethanolic, water and propylene glycol extracts obtained from *A. vulgaris* (GAE, GAW, GAP, respectively) on skin hydration levels in vivo revealed that TEWL values measured on SLS-irritated human skin of 18 volunteers by Tewameter^®^ TM 210 were back to normal. Furthermore, SCH (stratum corneum hydration) parameters measured using Corneometer^®^ CM 820 proved action towards skin hydration level after 3 days of application [35].

## 4. Active Compounds Responsible for Skin Benefits

In *Alchemilla* species with skin-conditioning effects, a variety of compounds have been identified as key factors. These are predominantly polyphenolic compounds, which align with the biological activities observed [6,58]. The chemical profile of *Alchemilla* species is rich and complex, and a full phytochemical inventory is beyond this review’s scope; however, notable active compounds include:

- Ellagitannins and gallotannins: These high-molecular-weight polyphenols (e.g., agrimoniin, pedunculagin) are abundant in *Alchemilla* and largely responsible for their astringent, antimicrobial, and antioxidant properties [47]. For instance, agrimoniin (isolated from *A. vulgaris* and *A. mollis*) has demonstrated anti-inflammatory effects by inhibiting NF-κB activation in immune cells [47,48]. Pedunculagin, found in *A. vulgaris* and *A. mollis* leaves, showed significant anti-acne activity; Kim et al. [59] reported that pedunculagin inhibits *Propionibacterium acnes*-induced inflammation and reduces sebum oxidation. These tannins also chelate metals and precipitate proteins, explaining the wound-healing benefit (they can form a protective layer on wounds and contract tissues, aiding clotting and tissue firming).
- Flavonoids: *Alchemilla* species contain various flavonoids (flavonols, flavones) such as quercetin and its glycosides, kaempferol glycosides, and apigenin. Apigenin has been identified in *A. vulgaris* and *A. caucasica* aerial parts [60,61]. This flavonoid is known to protect against UV-induced skin damage, stimulate collagen synthesis, and aid in conditions like psoriasis and vitiligo [62]. A recent comprehensive review by Majma Sanaye et al. [62] highlighted apigenin’s dermatological benefits (anti-photoaging, wound healing, anti-cancer). Quercetin-3-*O*-*β*-glucuronide from *A. vulgaris* was shown to be a potent collagenase inhibitor (as mentioned, it can preserve skin elasticity by blocking collagen breakdown) [38,39]. Flavonoids also contribute strongly to *Alchemilla*’s antioxidant activity. Isoquercitrin, juglanin, and other flavonols reported in *Alchemilla* likely synergize to scavenge free radicals and reduce oxidative stress in skin cells.
- Phenolic acids: Gallic acid and ellagic acid are common in *Alchemilla*. Ellagic acid (3.4 mg/g in an ethanolic extract of *A. vulgaris* leaves) was quantified by Tasić-Kostov et al. [35]. Ellagic acid is a known antioxidant and also a tyrosinase inhibitor, contributing to skin-lightening effects. Gallic acid and its derivatives provide antimicrobial action. In an analysis of *A. vulgaris*, gallic acid content correlated with strong DPPH scavenging [47]. These phenolics, while not unique to *Alchemilla*, bolster their overall efficacy by targeting oxidative and microbial aspects of skin disorders.
- Proanthocyanidins: Oligomeric proanthocyanidins have been detected in some *Alchemilla* species. For example, Mandrone et al. [39] noted procyanidin-type compounds in *A. vulgaris* during their metabolomic identification of active compounds. Proanthocyanidins have anti-enzymatic activity (inhibiting collagenase and elastase) and contribute to vascular health in the skin (strengthening capillaries). Their presence could explain *Alchemilla* extracts’ use in anti-redness cosmetics and as firming agents.
- Trace elements: An interesting aspect of *Alchemilla* is its bioaccumulation of certain minerals. *A. velebitica* was found to be a rich source of trace elements like zinc, manganese, and selenium in its leaves and roots [63]. Zinc (18–54 mg/kg in *A. velebitica*) is essential for skin repair and has anti-acne properties when applied topically [63]. The presence of these elements suggests that *Alchemilla* extracts might deliver not only organic compounds but also micronutrients that benefit skin structure and immune defense. However, these findings come from one study of a specific species; it indicates potential, but further analyses of other species are needed.

Notably, many of the above compounds work in concert. For example, *A. vulgaris* extracts contain both ellagitannins and flavonoids; the ellagitannins may provide immediate antibacterial astringency, while flavonoids modulate inflammation and oxidative stress over time. This synergistic complexity is likely why whole-plant extracts often outperform isolated compounds in efficacy—a phenomenon observed in several studies where fractions of *Alchemilla* lost some activity when purified too far [47]. It also underlines the wisdom of traditional medicine using whole extracts or decoctions.

On the other hand, the complexity of *Alchemilla*’s phytochemistry poses challenges: standardizing an extract for consistent bioactivity requires careful control of harvesting (phenolic content can vary with geography and season). Moreover, high-tannin extracts can be irritating if used at excessive concentrations (tannins may cause dryness or rare allergic reactions) [64]. Formulating these extracts in modern delivery systems (nano-formulations, appropriate pH buffers) can improve bioavailability and reduce potential irritation [49]. Studies are starting to explore such approaches—for instance, encapsulating *Alchemilla* extract in lipid nanoparticles to enhance skin penetration of its active compounds [65].

In summary, *Alchemilla*’s skin benefits stem from a diversity of phenolic constituents, with tannins and flavonoids playing the main role. Each compound class tackles a different aspect of skin pathology, making *Alchemilla* extracts inherently multi-functional. Future research that isolates these compounds can further confirm their individual roles (e.g., confirming which specific tannin most strongly inhibits *C. acnes* growth or which flavonoid best stimulates collagen). Importantly, any development of *Alchemilla*-based therapeutics should consider preserving the natural synergy of its compounds to maximize efficacy.

## 5. Various Cosmetic Formulations and Preparations

Aiming to develop consumer-acceptable products for topical application, in recent years, researchers have attempted to determine which formulations are the most optimal for the effective release of the active factors contained in *Alchemilla* extracts by analyzing such parameters as hardness, consistency or cohesiveness. Due to understanding the mechanisms of skin penetration at the cellular and molecular level, scientists are increasingly examining the impact of advanced technologies, such as nanotechnology, in order to more effectively release and deepen the absorption of active compounds. Inorganic and polymeric nanoparticles (for example, titanium dioxide, zinc oxide or gold nanocarriers) play an important role in this process [65].

A noteworthy example is hydrogels, which are polymeric materials that have the ability to absorb large amounts of water without losing their structure. Both the degree of swelling and the mechanical properties of hydrogels can be changed by selecting the synthesis conditions and monomers so that they respond to external conditions (such as pH temperature) in a desired manner. Hydrogels are employed as vehicles for the modified release of active ingredients [66]. Thermosensitive hydrogels such as poly(N-isopropylacrylamide), p(NIPAM), and poly(Nisopropylacrylamide-co-2-hydroxypropyl methacrylate), p(NIPAM-HPMet) were used for the study conducted by [66]. The ability to release ellagic acid from various hydrogels obtained from the aboveground parts of *A. vulgaris* has been studied using FTIR and scanning electron microscope methods. The experiment indicated that p(NIPAM-HPMet) showed better incorporation and release at 37 °C of ellagic acid (98.87%) and *A. vulgaris* extract (96.45%) compared to p(NIPAM) [66].

As reported in Tasić-Kostov et al. [35], ethanolic, water and propylene glycolic extract obtained from *A. vulgaris* were tested for wound healing and antioxidant properties in combination with gel-based on Carbopol^®^ Ultrez. The most favorable effect on wound healing in vitro was obtained for gel-containing propylene glycolic extract. Values obtained using textural assays demonstrated promising mechanical properties of the gels. Thus, they can be considered vehicles intended for wound treatment with topical application of *A. vulgaris*. It can be concluded that propylene glycol is an effective permeation enhancer in gel formulations and a desirable solvent for the production of herbal extracts.

Another kind of formulation used for topical treatment of *A. vulgaris* extract is an ointment. In the study carried out by Choi et al. [37], the application of ointment, including the herbal mixture on the dorsal skin wounds of mice (wound model on Seven-week-old male BALB/c mice) revealed significant wound healing activity proceeding faster than therapy with fusidic acid [37].

A survey on the effect of using various formulations differing in polarity (such as ointments and creams) on the antibacterial and antifungal activity of *A. pedata* extract was performed by Taddese et al. [33]. Seven formulations (form) were tested, each containing 10% of the extract, namely: form 1: sodium laurylmonosterin cream base; form 2: macrogol cream base; form 3: macrogol blend ointment base; form 4: simple ointment base; form 5: white petrolatum; form 6: polyethylene glycol bases (PEG 400:PEG 2000, 1:1) and form 7: polyethylene glycol bases (PEG 400:PEG 2000, 1:3). The test revealed that releases of the active ingredients from hydrophilic bases were higher and polyethylene glycol-based formulations were more active than formulations prepared with hydrophobic bases. No release has been observed from the simple ointment and white petrolatum (forms 4 and 5). This may be related to the fact that the agar medium is hydrophilic, which results in the adhering of hydrophobic components to the medium for a longer period than they diffuse into the hydrophilic surrounding environment [33].

Another aspect of the application of extracts obtained from the *Alchemilla* genus is the possibility of using them orally to achieve positive health-promoting effects, including activity within the skin tissues. Mansour et al. [67] demonstrated the efficacy of dietary supplementation of *A. vulgaris* powder in the diet of Nile tilapia (*Oreochromis niloticus*) fish in a dose-dependent manner. Oral application of *A. vulgaris* showed antioxidant activity and related increase of antioxidant biomarkers (catalase—CAT, superoxide dismutase—SOD, total antioxidant—TAC, and reduced glutathione—GSH as well as the reduction in myeloperoxidase (MPO) and malondialdehyde (MDA) levels. Nevertheless, further studies on the safety of use in the human body are required.

## 6. Safety of Application

An essential aspect, not only from the point of view of increasingly conscious consumers but also from the point of view of cosmetics manufacturers, is the assessment of the safety level of products. Accordingly, Boroja et al. [26] performed a biocompatibility analysis of *A. vulgaris* extract using a cell survival assay (immortalized murine BalbC-3 T3 fibroblasts and human normal HaCaT keratinocytes). No significant differences were observed in cell survival between the control group and groups treated with extracts [26]. Moreover, a study performed by Dos Santos Szewczyk et al. [8] employing the MTT assay reported that the butanol fraction of *A. acutiloba* methanolic extract is not cytotoxic towards GMK cells (CC_50_ value was 1000 µg/mL).

## 7. Comparison with Other Dermatological Medicinal Plants

In our opinion, it is useful to compare *Alchemilla* with other plant species known for treating skin inflammation and disorders to understand the novelty and impact of this genus in the broader context of herbal dermatotherapy. Many medicinal plants have similar phytochemical profiles (rich in polyphenols, tannins, terpenoids, etc.) that provide dermatological benefits such as anti-inflammatory, wound-healing, and antimicrobial effects. Below, we discuss a few notable examples and how *Alchemilla* relates to them:

- *Aloe vera* (L.) Burm.f.: *Aloe vera* gel is a popular natural remedy for skin hydration, wound healing, and mild psoriasis. *Aloe* spp. contain polysaccharides and glycoproteins that soothe irritation and promote tissue repair. Clinical studies have found that topical *Aloe vera* can improve mild to moderate psoriasis plaques, with anti-inflammatory and moisturizing effects [64]. Compared to *Aloe*, *Alchemilla* extracts are more astringent (due to tannins) but similarly anti-inflammatory. Both can reduce skin redness and aid wound closure, though via different mechanisms: *Aloe* mainly provides moisture and growth factors, whereas *Alchemilla* provides antioxidants and antimicrobial tannins. Interestingly, a review of natural psoriasis treatments noted that herbal remedies like *Aloe vera*, *Indigo naturalis*, and *Mahonia aquifolium* have yielded positive outcomes in reducing psoriatic lesions [64]. *Alchemilla* is not yet commonly cited in such contexts, highlighting its under-recognition. Given its properties, *Alchemilla* could complement or enhance treatments like *Aloe*, potentially offering both soothing and antiseptic actions in one.
- *Calendula officinalis* L. (Marigold) and *Matricaria chamomilla* L. (Chamomila): These are classic skin-healing herbs used for anti-inflammatory and soothing purposes in eczema, diaper rash, etc. *Calendula* flowers contain triterpenoids and flavonoids that increase wound healing and reduce dermatitis; *Chamomila* contains bisabolol and chamazulene that calm irritation. Both have established topical uses for skin regeneration. *Alchemilla* shares the wound-healing promotion seen with *Calendula*—for example, *A. mollis* and *A. persica* extracts increased wound tensile strength in animals compared to how *Calendula* ointment accelerates wound closure [20,68,69]. *Chamomila* and *Alchemilla* both have notable antioxidant activity; however, *Alchemilla*’s antimicrobial activity is generally greater, owing to its higher tannin content, while *Chamomila* is milder in that regard. An advantage of *Alchemilla* is its multifaceted action (antioxidant, antimicrobial, and astringent), whereas these other herbs are primarily anti-inflammatory and moisturizing. Thus, *Alchemilla* could potentially replace or complete *Calendula*/*Chamomila* in situations where infection risk is present along with inflammation (such as acne or infected eczema).
- Indigo naturalis: A Traditional Chinese Medicine remedy (Qing Dai) used in psoriasis. Indigo naturalis (derived from *Strobilanthes formosana* S.Moore or related indigo plants) has shown potent anti-psoriatic effects in clinical studies. It reduces inflammation and excessive keratinocyte proliferation when applied topically (the active compound—indirubin, modulates aryl hydrocarbon receptors in the skin) [64]. *Alchemilla* extracts have not been tested in psoriasis patients yet, but they do mitigate some pathological factors (e.g., oxidative stress). Unlike Indigo naturalis, which can cause a temporary blue staining of skin, *Alchemilla* extracts are tannin-rich and tend to have a brown hue and astringent feel. This difference might affect formulation preferences, but it also indicates that *Alchemilla* could be cosmetically acceptable in products if appropriately prepared.
- *Berberis aquifolium* Pursh (Oregon grape): This plant’s root extract is an approved topical remedy for psoriasis, rich in isoquinoline alkaloids (e.g., berberine) that have anti-inflammatory and anti-proliferative effects on keratinocytes. *Berberis* creams have demonstrated significant improvements in psoriatic scaling and inflammation in clinical trials [64]. *Alchemilla* does not contain alkaloids like berberine, but its ellagitannins might achieve some similar anti-inflammatory outcomes via antioxidant pathways. Both *Berberis* and *Alchemilla* have antimicrobial activity that is beneficial for preventing secondary infection in chronic dermatitis. Given *Berberis*’ success, *Alchemilla* could be considered a candidate for similar development—especially because it also inhibits tyrosinase (offering an added benefit for managing post-inflammatory hyperpigmentation, where *Berberis* mainly targets inflammation).
- *Withania somnifera* (L.) Dunal (Ashwagandha): This Ayurvedic herb contains withanolides like withaferin A, which have been extensively studied for skin-related pharmacological effects. Withaferin A is a steroidal lactone that exhibits strong anti-inflammatory, anti-proliferative, and antioxidant activities in dermatological disease models [70]. It has shown efficacy against psoriasis and even skin cancers by modulating immune responses and inducing apoptosis in abnormal cells [70]. *Alchemilla* possess some comparable properties—for instance, both *Alchemilla* tannins and withaferin A can reduce inflammatory mediators and oxidative stress. However, *Alchemilla* does not contain a single compound similar to withaferin; instead, it delivers a synergistic mix of polyphenols. Ashwagandha is also a more researched plant (with numerous clinical trials, e.g., in atopic dermatitis), underscoring the need for deeper *Alchemilla* investigations. Nonetheless, the broad-spectrum dermatological effects of *Alchemilla* extracts (antioxidant, antimicrobial, etc.) parallel Ashwagandha’s multi-modal actions, suggesting that *Alchemilla* could likewise be developed into topical formulations for inflammation-driven skin conditions [70].

In summary, *Alchemilla* stands out as a multi-purpose dermatological herb, much like other known medicinal plants, but it remains less studied and used. Its antioxidant and antimicrobial potency are comparable to or exceeding some well-known skin herbs, and its wound-healing and anti-inflammatory effects complement those of staples like *Aloe* and *Calendula*. The comparison emphasizes that *Alchemilla* has a valuable role to play—it could be considered alongside Ashwagandha for anti-inflammatory uses, or alongside *Berberis* and Indigo naturalis for psoriasis, and as an alternative to *Calendula* in wound and rash remedies. The key novelty of *Alchemilla* is the combination of diverse activities in one genus. Future direct comparative studies (e.g., *Alchemilla* vs. *Calendula* in wound healing efficacy or *Alchemilla* vs. *Berberis* in psoriasis models) would help position this genus in herbal dermatology. Current data already justify integrating *Alchemilla* extracts into modern skin care products, especially given the growing trend of formulating multi-herb complexes for synergistic effects [2]. *Alchemilla*’s inclusion could enhance the impact of such formulations by contributing strong antioxidant and antimicrobial components.

## 8. Discussion of Key Findings and Practical Implications

Based on the above results and comparisons, several general points emerge regarding how *Alchemilla* extracts can be optimally utilized and what gaps in our knowledge remain:

- Optimal extraction methods: The effectiveness of *Alchemilla* extracts clearly depends on the extraction solvent and method. Generally, hydroalcoholic extracts (e.g., 50–70% ethanol or methanol) and medium-polarity fractions (ethyl acetate, butanol) contain the highest levels of active polyphenols and tend to exhibit the strongest antioxidant and enzyme-inhibiting activities. For instance, partitioning a methanol extract of *A. acutiloba* yielded an ethyl acetate fraction that concentrated most of the antioxidant tannins. In contrast, purely aqueous extracts can be less potent in some assays but still retain significant activity (as seen with *A. mollis* water extracts effective against *S. aureus*). Completely non-polar extracts (hexane) sometimes yield specific actives (like the potent DPPH scavenger in *A. ellenbergiana*), but generally, a mix of water and alcohol is recommended to extract a broad spectrum of actives (tannins, flavonoids, and phenolic acids). Maceration or reflux extraction is commonly used in studies; however, modern techniques like ultrasonic-assisted extraction could improve yield and skin delivery of *Alchemilla* compounds. In practical terms, for topical formulation development, a glycerol- or propylene glycol-containing extract might offer an optimal compromise, harnessing both hydrophilic and lipophilic constituents. The data suggest that no single solvent captures all active compounds, so sequential extraction (graduating from non-polar to polar) might be employed to create a full-spectrum *Alchemilla* extract for maximal efficacy.
- Efficacy of study models: The review encompassed a range of experimental models—from test-tube antioxidant assays to animal wound-healing models—each providing different insights. In vitro assays (DPPH, FRAP, enzymatic inhibition) are useful for screening antioxidant or anti-enzyme potential, but they simplify complex skin physiology. Cell culture models, such as fibroblast scratch assays and keratinocyte inflammation tests, offer more biological relevance by showing how extracts interact with living cells (e.g., promoting cell migration or reducing inflammatory markers). These were effective in demonstrating *Alchemilla* wound-healing stimulation and anti-inflammatory impact on skin cells. The most convincing evidence comes from in vivo models: rodent studies of wound healing illustrated that *Alchemilla* extracts significantly accelerate actual tissue repair and improve the biomechanical strength of healed skin. Such models capture the integrated effect of the extract on inflammation, cell proliferation, and remodeling phases of healing. Similarly, the in vivo melanoma model used for *A. vulgaris* provided crucial evidence that the extract can penetrate a biological system and exert anti-tumor effects. Going forward, ex vivo human skin models (e.g., skin explants) and clinical trials on patients are needed. The existing models have efficiently identified *Alchemilla* extract potential, but human trials will confirm its real-world effectiveness. For example, a small clinical study on an *Alchemilla* cream for acne or eczema would test whether the in vitro antimicrobial and anti-inflammatory effects translate to patient improvement. In summary, the combination of simple and complex models used so far has been effective in characterizing *Alchemilla* activities; the next step is to employ clinical and translational models, which is currently a gap.
- Most potent species and extracts: Among the species studied, certain ones stand out as particularly potent. *A. vulgaris* is the most extensively studied and shows well-rounded efficacy (good antioxidant, antimicrobial, enzyme inhibition, and wound-healing properties), making it a prime candidate for product development. However, *A. vulgaris* is not necessarily the most potent in each category. For example, *A. acutiloba* and *A. arvensis* exhibited exceptionally high antioxidant power, suggesting these species accumulate certain polyphenols to a higher level. *A. mollis* and *A. persica* exceed in wound-healing assays, indicating their chemical profile is especially suited for tissue repair stimulation. In terms of antimicrobial activity, *A. mollis,* with its low MICs against *S. aureus,* was notably potent, and *A. hybrida* showed strong antifungal activity with low MICs. These findings imply that while any of these *Alchemilla* species can be useful, selecting the right species-extract pair is key for a given application. For an antioxidant-rich serum, *A. acutiloba* or *A. alpina* extracts might provide the strongest effect, whereas, for an acne gel, *A. mollis* (antibacterial) combined with *A. vulgaris* (anti-inflammatory) could be ideal. It is also encouraging that less-common species (e.g., *A. hybrida*, *A. barbatiflora*) showed potent activities; this diversity means the genus as a whole is a rich resource. Future research could employ bioassay-guided fractionation to specify the most active species and standardize their extracts. Additionally, an interesting note is that some *Alchemilla* species untested so far are used in traditional medicine (like *A. hessii A. monticola*); investigating these could yield even more potent extracts or novel compounds. For now, based on current evidence, *A. vulgaris*, *A. mollis*, and *A. persica* are good all-around choices (with *A. vulgaris* having the advantage of more safety data historically), while *A. acutiloba* and *A. arvensis* are top performers in antioxidant assays, and *A. hybrida* is notable for antifungal efficacy.

In conclusion of this discussion, *Alchemilla* has demonstrated in vitro and in vivo efficacy that justifies further development, but the translation to clinical use will require addressing the above points: choosing the right extract method for the desired results, confirming efficacy in human models, selecting the most potent species or variety, and ensuring formulations maximize benefits while minimizing any side-effects.

## 9. Conclusions

In summary, *Alchemilla* (lady’s mantle) species exhibit a wide range of beneficial biological activities for skin health, confirming their traditional use in treating dermatological ailments. Key findings of this review highlight that *Alchemilla* extracts are rich in polyphenols with strong antioxidant properties, helping to neutralize free radicals and protect the skin from oxidative stress and aging. They also possess anti-inflammatory and astringent tannins that can soothe irritation, reduce redness, and promote the healing of minor wounds. Various *Alchemilla* extracts showed antimicrobial efficacy, particularly against Gram-positive bacteria implicated in skin infections and acne (e.g., *Staphylococcus aureus* and *Cutibacterium acnes*), as well as activity against certain fungi, which supports their use in preventing or treating infected wounds and rashes. Preliminary studies even indicate anti-tumor potential, with *A. vulgaris* extracts inhibiting melanoma cell growth, though more research is needed in this area.

Practical applications of these findings are already emerging: *Alchemilla* can be incorporated into natural skincare and cosmetic products as an active ingredient for anti-aging (due to its collagenase/elastase inhibitory effects and antioxidants), for acne-care formulations (providing a botanical antibacterial and anti-inflammatory action without the side effects of synthetic agents), and for wound-healing ointments or sprays (to speed up repair and keep wounds clean). The best-studied species, *A. vulgaris*, along with *A. mollis* and *A. persica*, appear particularly promising for such uses. Importantly, this review underlines that among nearly 800 species, just a few have been studied—indicating a rich untapped potential. Future research should focus on exploring these less-studied *Alchemilla* species (e.g., ethnomedicinal plants like *A. hessii* and *A. monticola*), as they may yield new bioactive compounds or even greater efficacy in skin applications.

Continued investigation is also encouraged to fully clarify the mechanisms of action of *Alchemilla* extracts at the molecular level (for example, how exactly they modulate inflammatory pathways or influence skin microbiome balance). Rigorous safety evaluations and clinical trials will be important to translate the in vitro and in vivo findings into approved medical or cosmetic products. This includes confirming that *Alchemilla*’s tyrosinase inhibition is safe for long-term use (ensuring no cytotoxicity to melanocytes) and establishing optimal dosing regimens. Given the current evidence, *Alchemilla* extracts appear to have a favorable safety profile and a low risk of side effects, especially in topical use, but clinical validation will build confidence for consumers and healthcare providers.

In conclusion, *Alchemilla* species represent a versatile and potent natural resource for dermatological therapy and skincare. They combine antioxidant, anti-inflammatory, antimicrobial, and wound-healing activities in one package, a synergy that modern single-target drugs often lack. Using *Alchemilla*-based preparations could reduce reliance on synthetic chemicals (like corticosteroids or antibiotics) in managing certain skin conditions, aligning with the growing demand for gentler, plant-based treatments. To fully realize this potential, future studies should aim to broaden the spectrum of species studied, standardize extract preparations for consistency, and pursue clinical trials in conditions such as acne, eczema, psoriasis, and wound care.

## Figures and Tables

**Table 1:** Biological activities of *Alchemilla* species are valuable for dermatological diseases and skin care (presented on the basis of original studies published until December 2024).

Biological Activity	*Alchemilla* Species	Part of the Plant	Extraction Used	Study Model	**Results**	**Ref.**
antioxidant	*A. acutiloba*	aerial parts and roots	60% methanol extract and its diethyl ether, ethyl acetate and n-butanol fractions	in vitro; DPPH^•^, ABTS^•+^, chelating activity	DPPH^•^—IC_50_ = 51.42–8.83 µg/mL; ABTS^•+^—IC_50_ = 24.82–1.42 µg/mL; chelating activity—44.12–11.43 µg/mL	[8]
	*A. alpina*	aerial parts	methanol extract	in vitro; DPPH^•^	% Inhibition = 45.4–94.4%	[9]
	*A. arvensis*	leaves	methanol, hexane, acetone, water extracts	in vitro; DPPH^•^	IC_50_ = 97.72–4.86 µg/mL	[10]
	*A. barbatiflora*	aerial parts	90% methanol extract and its n-hexane, chloroform and water fractions	in vitro; DPPH^•^, SOD (superoxide radical scavenging), phosphomolibdenum-reducing antioxidant power (PRAP), ferric-reducing antioxidant power (FRAP)	% Inhibition: DPPH^•^—18.6–97.17%; SOD—9.80–83.34%. Reducing power: PRAP—0.355–1.516; FRAP—15.76–93.46 mg BHAE/g extract	[11]
	*A. ellenbergiana*	aerial parts	hexane, ethanol, methanol extracts	in vitro; DPPH^•^	IC_50_ = 7.1–243.6 µg/mL	[12,13]
	*A. glabra*	aerial parts	80% acetone (in 0.2% formic acid) extract	in vitro; ORAC, TRAP, HORAC	ORAC—1337 μmol TE/g; TRAP—1815 μmol TE/g; HORAC—1999 μmol GAE/g	[14]
	*A. hybrida*		ethanol extract	in vitro; DPPH^•^	IC_50_ = 0.082 mg/mL	[15]
	*A. jumrukczalica*	leaves	methanol extract	in vitro; DPPH^•^	IC_50_ = 12.09 µg/mL	[16]
	*A. lithophila*	aerial parts	methanol, water and hexane extracts	in vitro; DPPH^•^, ABTS^•+^	% Inhibition: DPPH^•^—10.51–75.38%; ABTS^•+^—10.56–70.67%	[17]
	*A. mollis*	leaves	50% ethanol extract	in vitro; DPPH^•^, ABTS^•+^, ROS (reactive oxygen species)	DPPH^•^—IC_50_ = 42.4 µg/mL; ABTS^•+^—IC_50_ = 7.8 µg/mL; 10 and 100 µg/mL—22.3% and 48.0% decreases in ROS level	[18]
		herb	hexane, ethyl acetate, methanol, 50% methanol, deodorized water extracts	in vitro; DPPH^•^, ABTS^•+^	DPPH^•^—IC_50_ = 0.26–0.15 mg/mL; TEAC—0.90–1.55 mM/L/Trolox	[19]
		aerial parts and roots	80% methanol extract	in vitro; DPPH^•^	IC_50_ = 39.4 (aerial parts)–114.6 (roots) µg/mL	[20]
		stalks	10–90% ethanol extract	in vitro; DPPH^•^, ABTS^•+^, FRAP, CUPRAC	DPPH^•^—21.50–247.58 mmol TE/dm^3^; ABTS^•+^—27.10–308.44 mmol TE/dm^3^; FRAP—67.91–382.78 mmol TE/dm^3^; CUPRAC—79.72–363.79 mmol TE/dm^3^	[21]
		aerial flowering parts	methanol extract and its ethyl acetate, petroleum, chloroform, water fractions	in vitro; DPPH^•^	IC_50_ = 9.8–>200 µg/mL	[22]
	*A. persica*	aerial parts and roots	80% methanol extract	in vitro; DPPH^•^, TBARS	DPPH^•^—IC_50_ = 0.055 M (aerial parts), 0.151 M (roots); TBARS—5.9 nmol/mL (aerial parts), 19.08 nmol/mL	[23]
	*A. sericata*	aerial parts	95% hexane extract	in vitro; DPPH^•^	IC_50_ = 185 µg/mL	[24]
	*A. vulgaris*	herb	aqueous and 70% ethanolic extracts	in vitro; DPPH^•^	% Inhibition: DPPH^•^—80.71–87.95%	[25]
		aerial parts and roots	methanol extract	in vitro; DPPH^•^, ABTS^•+^, ^•^OH, total antioxidant activity (TAA), metal chelation, reducing capacity, inhibition of lipid peroxidation	DPPH^•^—IC_50_ = 5.96–11.86 µg/mL; ABTS^•+^—IC_50_ = 14.80–32.49 µg/mL; ^•^OH—IC_50_ = 13.06–18.44 µg/mL; lipid peroxidation—IC_50_ = 31.91–475.13 µg/mL; TAA—265.62–316.47 mg AA/g of extract; reducing—632.99–607.52 mg Trolox/g of extract	[26]
		aerial parts	80% methanol, 70% ethanol, 70% ethylacetate, water extracts	in vitro; DPPH^•^, ABTS^•+^, CUPRAC, FRAP, phosphomolibdenum and metal chelating assays	DPPH^•^—89.25–502.56 mg TE per g extract; ABTS^•+^—37.50–174.05 mg TE per g extract; CUPRAC—78.56–283.16 mg TE per g extract; FRAP—3240.09–8745.31 mg AAE per g of extract; Phosphomolibdenum—0.53–2.22 mmol TE per g extract; metal chelating—37.96–42.58 mg EDTAE per g extract	[27]
		aerial parts	50% ethanol extract	in vitro; DPPH^•^, metal chelating assay	% Inhibition: DPPH^•^—71.8; metal chelating—84.6%	[28]
		leaves	80% ethanol and water extracts	in vitro; DPPH^•^	% Inhibition: 131.74%	[29]
		roots	50% ethanol extract	in vitro; ABTS^•+^, FRAP	ABTS^•+^—68.21 mmol TE/g DW; FRAP—40.12 mmol of TE/g DW	[30]
	*A. xanthochlora*	leaves	hexane, chloroform, ethylacetate, methanol, water extracts	in vitro; DPPH^•^	0.23–2.49 g DPPH/g DE	[31]
anti-inflammatory	*A. acutiloba*	aerial parts and roots	60% methanol extract and its diethyl ether, ethyl acetate and n-butanol fractions	in vitro; COX1, COX2	% Inhibition: COX1—31.27–83.14%; COX2—43.65–90.93	[8]
	*A. mollis*	aerial parts and roots	80% methanol extract	in vivo; Whittle method	% Inhibition: 5.3–30.6%	[20]
				in vivo; human red blood cell (HRBC) membrane stabilization method	IC_50_ = 1.22–1.24 mg/mL	[32]
	*A. persica*	aerial parts and roots	80% methanol extract	in vivo; Whittle method	% Inhibition: 3.6–26.6%	[20]
				in vivo; human red blood cell (HRBC) membrane stabilization method	IC_50_ = 1.52–1.82 mg/mL	[32]
	*A. vulgaris*	aerial parts and roots	methanol extract	in vitro; COX-1 and COX-2	% Inhibition: COX-1—44.1–44.4%; COX-2—40.4–63.6%	[26]
antibacterial	*A. arvensis*	leaves	methanol, hexane, acetone, water extracts	agar diffusion and micro-broth dilution methods (*Staphylococcus aureus*, *Escherichia coli*, *Pseudomonas aeruginosa*, *Shigella sonnie*)	MIC values 6.3–25.0 mg/mL	[10]
	*A. mollis*	herb	hexane, ethyl acetate, methanol, 50% methanol, deodorized water extracts	agar dilution method (*S. aureus*, *E. coli*, *P. aeruginosa*, *Enterococcus faecalis*)	MIC values 0.5–7.5 mg/mL MC values 5–10 mg/mL	[19]
		aerial parts and roots	80% methanol extract	microbroth dilution method (*S. aureus*, *E. faecalis*, *B. subtilis*, *E. coli*, *P. aeruginosa*)	MIC values 5–10 mg/mL	[32]
	*A. pedata*		petroleum ether, chloroform, acetone and methanol extracts	agar well diffusion method (*E. coli*, *P. aeruginosa*, *S. aureus*)	MIC values 12.5–20.16 mg/mL	[33]
	*A. persica*	aerial parts and roots	80% methanol extract	microbroth dilution method (*S. aureus*, *E. faecalis*, *B. subtilis*, *E. coli*, *P. aeruginosa*)	MIC values 2.5–10.0 mg/mL	[32]
	*A. vulgaris*	aerial parts	50% ethanol/6% glycerin	agar well diffusion method (*S. epidermis*, *P. acnes, P. granulosum*)	inhibitory zone 0–13 mm	[34]
		aerial parts and roots	methanol extract	microdilution method (*Micrococcus lysodeikticus*, *Salmonella typhimurium*, *Bacillus subtilis*, *E. faecalis*, *E. coli*, *Klebsiella pneumoniae*, *P.* *aeruginosa*, *Bacillus mycoides*, *Azotobacter chroococcum*)	MIC values 0.156–0.625 mg/mL	[26]
antifungal	*A. arvensis*	leaves	methanol, hexane, acetone, water extracts	micro-broth dilution method (*Epidermophyton floccosum*, *Candida albicans*)	MIC values 0.78–12.5 mg/mL	[10]
	*A. hybrida*		ethanolic extract	microdilution method	MIC values 0.104–1.667 mg/mL	[15]
	*A. mollis* *A. persica*	aerial parts and roots	80% methanol extract	microbroth dilution method (*Candida albicans*)	MIC values 2.5–10.0 mg/mL	[32]
	*A. vulgaris*	aerial parts and roots	methanol extract	microdilution method (*Phialophora fastigiata*, *Penicillium canescens*, *Trichoderma viride*, *Trichoderma longibrachiatum*, *Aspergillus brasiliensis*, *A. glaucus*, *Fusarium oxysporum*, *Alternaria alternata*, *Doratomyces stemonitis*, *C. albicans*)	MIC values 2.5–20.0 mg/mL	[26]
wound-healing	*A. mollis* *A. persica*	aerial parts and roots	80% methanol extract	in vivo; Male Swiss albino mice and Sprague-Dawley rats	tensile strength value 33.3–39.3%; contraction value 43.5–51.4%)	[20]
	*A. vulgaris*	herb	70% ethanol, water, 80% propylene glycol extracts	in vitro; scratch assay with L929 fibroblasts; in vivo	wound healing potential	[35]
			3% extract in glycerine	in vivo		[36]
			herbal mixture	in vivo; in vitro scratch assay		[37]
anti-aging and skin-lightening (enzyme inhibitory)	*A. vulgaris*	herb	aqueous and 70% ethanolic extracts	in vitro; tyrosinase inhibition	% Inhibition: 71.55%	[25]
		herb	50% methanol extract	in vitro; collagenase inhibition	% Inhibition: 40.0%	[38,39]
		herb	50% methanol extract	in vitro; elastase inhibition	% Inhibition: 12.0%	[40]
		aerial parts	80% methanol, 70% ethanol, 70% ethylacetate, water extracts	in vitro; tyrosinase inhibition	Inhibition: 73.68–79.84 mg KAE per g extract	[27]
anti-tumor activity (anti-melanoma)	*A. vulgaris*	aerial parts	ethanol extract	in vitro—mouse melanoma cell lines: B16 and B16F10; in vivo—syngeneic mouse melanoma model	significantly reduced tumor growth compared to controls	[41]

Abbreviations: DPPH^•^—2,2-diphenyl-1-picrylhydrazyl radical scavenging assay; FRAP—ferric reducing antioxidant assay; CUPRAC—cupric-reducing antioxidant capacity; ABTS^•+^—2,2′-azino-bis-(3-ethyl-benzothiazole-6-sulfonic acid) radical scavenging assay; COX-1/COX-2—cyclooxygenase-1/-2 inhibitory activity; MIC—minimum inhibitory concentration; ORAC—the oxygen radical absorbance capacity; TRAP—total reactive antioxidant potential; HORAC—hydroxyl radical antioxidant capacity assay; KAE—kojic acid; DW—dry weight.

## Data Availability

No new data were created or analyzed in this study. Data sharing is not applicable to this article.
